# Local structural distortion and electrical transport properties of Bi(Ni_1/2_Ti_1/2_)O_3_ perovskite under high pressure

**DOI:** 10.1038/srep18229

**Published:** 2015-12-16

**Authors:** Jinlong Zhu, Liuxiang Yang, Hsiu-Wen Wang, Jianzhong Zhang, Wenge Yang, Xinguo Hong, Changqing Jin, Yusheng Zhao

**Affiliations:** 1 HiPSEC, Department of Physics and Astronomy, University of Nevada, Las Vegas, Nevada 89154, USA; 2 Center for High Pressure Science and Technology Advanced Research (HPSTAR), 1690 Cailun Road, Shanghai, 201203, China; 3 High Pressure Synergetic Consortium (HPSynC), Geophysical Laboratory, Carnegie Institution of Washington, Argonne, IL 60439, USA; 4 Lujan Neutron Scattering Center, Los Alamos National Laboratory, Los Alamos, New Mexico 87544, USA; 5 Materials Science and Technology Division, Los Alamos National Laboratory, Los Alamos, New Mexico 87544, USA; 6 Mineral Physics Institute, Stony Brook University, Stony Brook, New York 11794, USA; 7 National Lab for Condensed Matter Physics, Institute of Physics, CAS, Beijing 100190, China

## Abstract

Perovskite-structure materials generally exhibit local structural distortions that are distinct from long-range, average crystal structure. The characterization of such distortion is critical to understanding the structural and physical properties of materials. In this work, we combined Pair Distribution Function (PDF) technique with Raman spectroscopy and electrical resistivity measurement to study Bi(Ni_1/2_Ti_1/2_)O_3_ perovskite under high pressure. PDF analysis reveals strong local structural distortion at ambient conditions. As pressure increases, the local structure distortions are substantially suppressed and eventually vanish around 4 GPa, leading to concurrent changes in the electronic band structure and anomalies in the electrical resistivity. Consistent with PDF analysis, Raman spectroscopy data suggest that the local structure changes to a higher ordered state at pressures above 4 GPa.

ABO_3_ Perovskites (where A and B are two cations in 12- and 6-fold coordination, respectively) are a large family of materials with many interesting properties and important technological applications, such as ferroelectricity, piezoelectricity, and high-temperature superconductivity, etc^1^,^2^,^3^,^4^,^5^,^6^. Local structural distortion in both the A and B sites is a general feature among perovskite-type systems and has a strong influence on their physical properties. For instance, the intrinsic TiO_6_ local bonding distortion and octahedral tilting in CaTiO_3_ perovskite-based materials can modify intermediate energy states within the band gap and associated photoluminescence emission profile^7^. The local structural distortion in perovskite manganites results in the abrupt changes in the zero-field heat conductivity near the ferromagnetic, charging-ordering, and phase transition^8^. The local structure distortion and related properties are sensitive to external stimuli such as temperature, pressure, strain, and applied fields. A typical example is the strain induced metal to insulator transition in perovskite manganities^8^. Application of external pressure is another effective way to tune material’s local structure and macroscopic properties without introducing impurities and/or chemical defects.

At ambient pressure, the orthorhombic-distorted Bi(Ni_1/2_Ti_1/2_)O_3_ perovskite is a multiferroic material exhibiting both magnetism and ferroelectricity^11^. The ferroelectric distortion is largely from the Ti^4+^ ions^12^ and the Bi^3+^ ions with 6*s*^2^ lone pair electrons^13^, whereas the interaction between 3*d* electrons of Ni^2+^ ions results in long-range magnetic ordering^13^. The local structure distortion is mainly due to ion radius mismatch between Ni^2+^ and Ti^4+^ ions and their disordered distributions in the octahedral site. Under high pressure, Bi(Ni_1/2_Ti_1/2_)O_3_ perovskite exhibits two isostructural phase transitions at ~2 GPa and ~15 GPa^11^. Based on Rietveld refinement of X-ray diffraction data, both transitions result from the discontinuous shifts of Bi ions. The distortion from the steric effect of Bi 6*s*^2^ lone pair is the source of ferroelectric distortion and is strongly coupled with the magnetic ordering^11^,^12^,^13^. To date, however, there has been no experimental determination of local structure distortion and phonon vibrational and electrical transport properties for Bi(Ni_1/2_Ti_1/2_)O_3_ perovskite under high pressure. These are important properties for materials exhibiting multiferroic behavior because magnetoelectric interaction between ferroelectricity and magnetism is typically bridged through the electron-phonon coupling. Therefore, a delicate local structure description and the possible local electric re-distribution will benefit the understanding of the magnetoelectric effect.

Pair distribution function (PDF) method has been proved to be an effective tool to determine differences between the local and the average crystal structures by using Fourier transform of the coherent and incoherent scattering, the so-called “total scattering” that include both Bragg peaks and diffuse scattering. PDF measures the distribution of every atom pair in a diffraction experiment, weighted by the scattering power of various atom pairs. It is capable of revealing atomic correlations from several Angstroms (local structure) to several hundred Angstroms (averaged structure). Typical applications include atomic-scale structural resolutions of nanoparticles, amorphous phases, and disordered crystalline materials^14^,^15^.

To better understand the local structural distortion and its relation with structural phase transition, local bonding and symmetry changes, and transport properties of Bi(Ni_1/2_Ti_1/2_)O_3_, and build up a delicate relationship between micro-local structure and macro-transport property, we have in this work carried out pair distribution function (PDF) analysis and Raman spectroscopy and electrical resistivity measurements under high pressure. Our results show that the local structure distortion is completely suppressed near 4 GPa, which correlates well with the isostructural phase transition around 2 GPa and abnormal changes in vibration modes, electronic band structure and electrical resistivity.

## Results and Discussions

**Figure 1:**
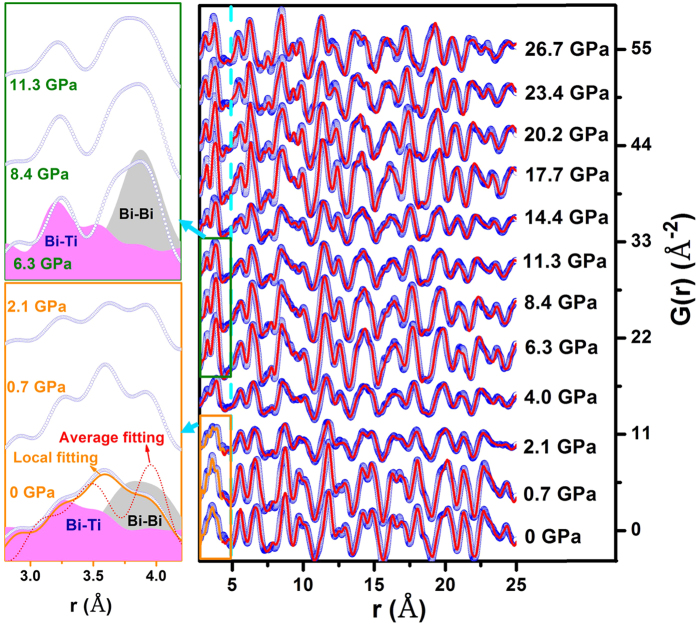
Local and average structure evolutions of Bi(Ni_1/2_Ti_1/2_)O_3_ under pressure. Pair correlation function, G(r), of Bi(Ni_1/2_Ti_1/2_)O_3_ obtained from Fourier transformation of X-ray total scattering data collected in a diamond anvil cell as a function of pressure up to 26.7 GPa. The blue dots are the experimental data, the red lines are from the PDFgui refinement for average structure, and the gold lines are the local structure refinement. The left panel shows the evolution of the local distortion at selected pressures. The dot red line is the average structure refinement. For the local structure at 0 GPa and unitive structure at 6.3 GPa, the partial PDFs for Bi-Bi and Bi-Ti pairs are plotted in gray shadow and magenta shadow, respectively.

**Figure 2:**
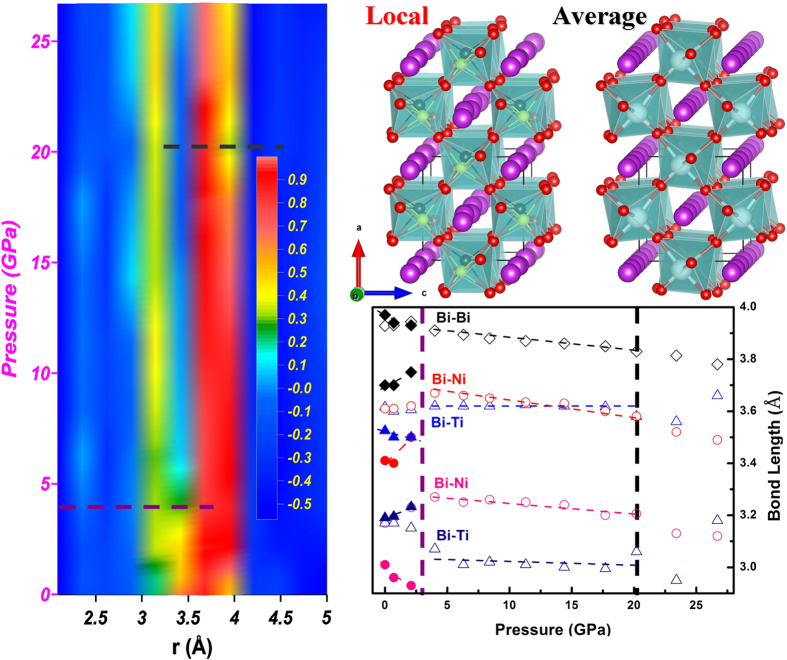
Structural parameters as a function of pressure. Left panel is a 2D drawing of the local PDFs at pressures up to 26.7 GPa, and the color scale bar represents the G(r) intensities. The right upper panel are schematic views of local and long-range (averaged) crystal structures of Bi(Ni_1/2_Ti_1/2_)O_3_ at ambient conditions. Ni^2+^ and Ti^4+^ ions in the local structure are represented by black and yellow balls, respectively, and in the average structure, they are shown by white balls as in ref. 11. The purple and red balls correspond to Bi^3+^ and O^2−^ ions, and TiO_6_ and NiO_6_ octahedrons are shown in light-cyan. Note that NiO_6_/TiO_6_ octahedrons are disordered in the average perovskite structure. The right lower panel plots Bi-Bi (diamonds), Bi-Ni (circles) and Bi-Ti (triangles) distances in the locally distorted structure (solid symbols) and long-range, averaged structure (open symbols). The vertical dash line at ~4 GPa indicates the vanishing of the local distortion, and the one at ~20 GPa is consistent with the second phase transition reported in ref. 11.

Figure 1 shows the experimental PDFs of Bi(Ni_1/2_Ti_1/2_)O_3_ as a function of pressure collected at room temperature. In the pressure range studied, the structural parameters and elastic bulk modulus obtained in this work are consistent with previously reported results^11^. For instance, Bi(Ni_1/2_Ti_1/2_)O_3_ crystallizes in *Pn*2_1_*a* space group with corner-shared NiO_6_ (TiO_6_) octahedrons as building blocks and the chains of Bi^3+^ ions along the *b* axis direction, as shown in Fig. 2. The lattice parameters of Bi(Ni_1/2_Ti_1/2_)O_3_ are *a* = 5.61 A°, *b* = 7.85 A°, and *c* = 5.58 A°; and the refined atomic positions of the averaged structure are listed in table I of ref. 11. At pressures below 4 GPa, the local structure distortion cannot well be described by the long-range, average crystal structure, as indicated by the left panels of both Figs 1 and 2. In the range of 1 to 5 angstrom, the local distortion results in the relatively shift of Bi atoms normal to the *b* axis and heavily distorted oxygen octahedrons, as demonstrated in Fig. 2 by the schematic views of the local and average structures at ambient conditions. As a result of the local distortion, the Bi atoms along the *b* axis form “zigzag” chains in two different orientations: the Bi atoms at the coordinate (x, 0, z) line up in the (10-1) crystallographic plane, while Bi atoms at (x, 0.5, z) line up in the (101) plane. Compared with the average structure, the off-center shifts of Bi atoms also lead to a shorter Bi-Bi distance in the (010) plane and longer Bi-Bi distance along *b* axis. The local distortion shifts the Bi atoms away from their ideal positions, forming two closely correlated peaks for the Bi-Bi pair which can be identified from the refinement, as plotted in the left panel of Fig. 1. By contrast, the long-range average structure only shows one broad peak for the same atom pair, as the partial PDF for Bi-Bi at 6.3 GPa in Fig. 1. The centers of the correlation peaks of Bi-Bi, Bi-Ni and Bi-Ti from the PDF analysis as a function of pressure are shown in the right lower panel of Fig. 2. All atomic bonds involving oxygen are not plotted due to its weak X-ray scattering factor and strong absorption by diamonds in the DAC. However, the distortion and tilting of the oxygen octahedrons, which are also tightly related to the structural distortion, need to be characterized by other methods, such as Raman spectroscopy, which will be discussed in the next paragraph. At pressures below 4 GPa, the local correlation between Bi and Ni ions (short and long) leads to shorter bond distances than those in the average structure, while no difference is found between local and average structures for the Bi-Ti distances as shown by open and solid triangles in Fig. 2. The different behaviors are mainly derived from the different electron configurations of Ni^2+^ and Ti^4+^ ions (i.e., with and without 3*d* electrons, respectively). According to the crystal field theory, structures with a transition metal in octahedral or tetrahedral coordination gain stability (or stabilization energy) for certain electronic configurations. In the case of Bi(Ni_1/2_Ti_1/2_)O_3_, Ni^2+^ would gain stability in an octahedral environment while Ti^4+^ does not. In this regard, the observed difference in the Bi-Ni interatomic distance can be attributed to the differences of the crystal field stabilization energy between a locally distorted and a more regular octahedral environment. This distinction can be preserved locally at pressures below 4 GPa when the crystal field effect cannot be completely suppressed. The disappearance of local structural distortion corresponds to the first iso-structural transition in ref. 11. There is an anomaly near 20 GPa in the atomic distance in Fig. 2, which corresponds to the second phase transition reported in ref. 11.

**Figure 3:**
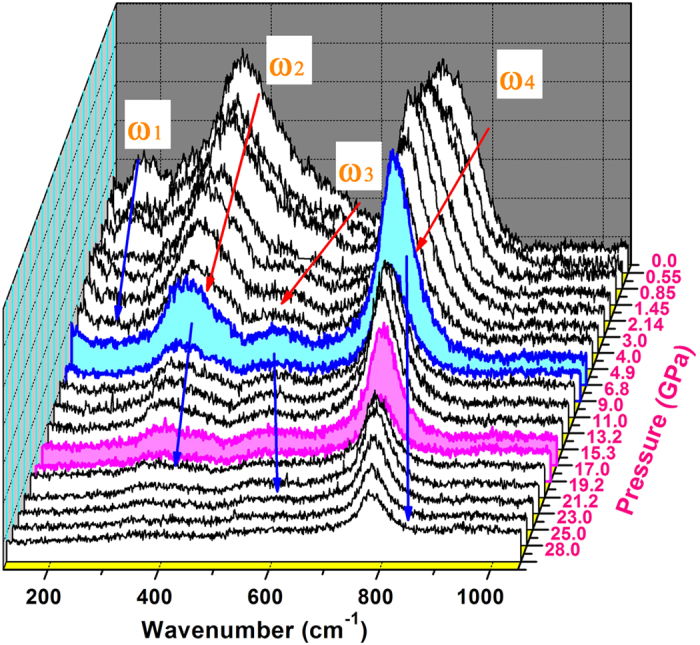
Vibrational modes of Bi(Ni_1/2_Ti_1/2_)O_3_. Raman spectra of polycrystalline Bi(Ni_1/2_Ti_1/2_)O_3_ as a function of pressure in the wave number range of 100 cm^−1^ to 1100 cm^−1^. The ω_1_ mode disappears at ~4 GPa, corresponding to the vanishing of local disorder. The ω_2_, ω_3_, ω_4_ modes change from red shift below 4 GPa to blue shift at higher pressures. At ~20 GPa, there is a discontinuous change in the Raman vibrational modes, consistent with the reported phase transition in ref. 11.

Raman vibrational modes are sensitive to local bonding and symmetry broken. Hence, they can provide useful information with regard to the local structure distortion. For the *Pn*2_1_*a* Bi(Ni_1/2_Ti_1/2_)O_3_, the Raman active modes can be expressed as Γ_optic_ = 17A_1_ + 18A_2_ + 17B_1_ + 17B_2_^9^. Figure 3 shows the Raman spectra of Bi(Ni_1/2_Ti_1/2_)O_3_ plotted as a function of pressure up to 28 GPa. At ambient pressure, four vibrational modes are observed and are located at 180 cm^−1^, 360 cm^−1^, 510 cm^−1^ and 720 cm^−1^, which are denoted as ω_1_, ω_2_, ω_3_, and ω_4_, respectively. As pressure increases, the ω_1_ mode shows a regular blue-shift up to 4 GPa, and then disappears at higher pressures. On the other hand, the ω_2_, ω_3_, and ω_4_ vibration modes all behave abnormally: red-shifts at pressures up to 4 GPa followed by blue-shifts upon further compression. Compared with a similar orthorhombic perovskite system previously reported by Iliev *et al.*^10^ and Wu *et al.*^16^, the ω_1_ vibration mode belongs to a crystal class of lower symmetry^16^, which could be activated when the local symmetry is broken due to either local structure distortion or defects. As pressure increases, the local symmetry in Bi(Ni_1/2_Ti_1/2_)O_3_ continues evolving toward the idealized *Pn*2_1_*a* structure until the ω_1_ mode becomes inactive near 4 GPa. The ω_2_ mode is assigned as B_2g_, ω_3_ as A_g_, both of which lie within the *xz* planes; and ω_4_ is a “mixed” mode of bending and rotation along the *y* direction^10^. The softening of these three vibration modes with increasing pressure up to 4 GPa can be tentatively understood from the local structure analysis discussed above. The locally distorted structure is driven by the mismatch of Ni and Ti atoms at the B site of ABO_3_ perovskite, which would introduce local tensile stress and strengthen the vibration modes. Accordingly, the suppression of local distortion by external pressure results the Raman modes shift to lower frequencies with increasing pressure below 4 GPa. Upon further compression, the observed shifts toward higher frequencies are due to pressure-induced bond shortening and lattice distortion. There are three main types of Raman vibrational modes, stretching, bending and rotational motions (Fig. 5 in ref. 10), which are sensitive to the distortion and tilting of the oxygen octahedron in Bi(Ni_1/2_Ti_1/2_)O_3_ perovskite. The “zigzag” chains formed by the Bi atoms along *b* axis exclude the stretching mode to be a driving force for the local distortion. On the other hand, the different shift directions of Bi atoms at (x,0,z) and (x,0.5,z) positions, as shown in Fig. 2, suggest that the local distortion comes from a combined effect of bending and rotation. For instance, the Raman bending mode can easily shift Bi atoms in (010) plane, all in the same direction, to form a “zigzag” chain along *b* axis in (10-1) crystal plane. The “zigzag” chain can further be rotated by the rotation mode when Bi atoms at (x, 0.5, z) positions are lined up in (101) plane, which is perpendicular to the “zigzag” chain in (10-1) plane. While a complete description of the local crystal configuration needs additional experimental data, such as single-crystal Raman spectroscopy, and proper theoretical modeling, we conclude that the ω_2_, ω_3_, and ω_4_ vibrations are bending and/or rotation modes, and they are less likely to be the stretching mode.

**Figure 4:**
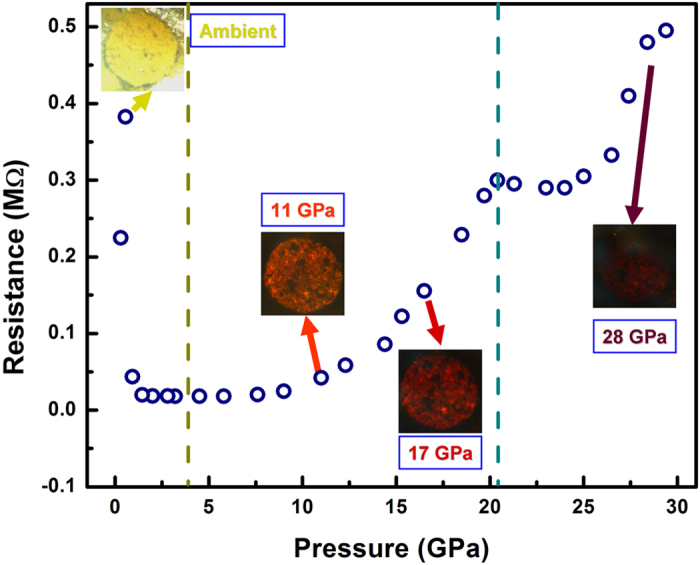
Electrical resistance of Bi(Ni_1/2_Ti_1/2_)O_3_ as a function of pressure. The resistivity data show a minimum near 4 GPa, where the local distortion vanishes, and a kink at around 20 GPa corresponding to the phase transition reported previously^11^. The insets are photos taken during Raman spectroscopy measurements, showing sample colors in the gasket chamber under different pressures. The sample at ambient pressure has a bright yellow color and is not optically transparent; with increasing pressure, the sample gradually becomes transparent and the sample color changes from red to dark red.

Suppression of the local structural distortion by applied external high pressure will simultaneously change the local electron hybridization and interaction, which would consequently modify the transport properties of Bi(Ni_1/2_Ti_1/2_)O_3_ perovskite. Figure 4 exhibits the electrical resistance measurement of polycrystalline Bi(Ni_1/2_Ti_1/2_)O_3_ at pressures up to 29.4 GPa, recorded during decompression in order to minimize the grain contact influence. Inspection of the data reveals a more than one order of magnitude drop in resistivity, from 0.382 MΩ at ambient conditions to 0.018 MΩ at ~4 GPa, indicating that the suppression of local distortion is in favor of narrowing band gap and enhanced electron transport. The resistance increases with pressure from 4 GPa to 29.4 GPa, which can primarily be attributed to the increased crystal packing density^17^. Consistent with high-pressure PDF analysis and Raman vibration data, there is a kink near 20 GPa in the resistance measurement, corresponding to an iso-structural phase transition as reported in ref 11.

In summary, PDF analysis of X-ray total scattering data indicates that the local structural distortion in Bi(Ni_1/2_Ti_1/2_)O_3_ perovskite is strongly suppressed by the external high pressure. Raman vibrational modes reveal that the bending and rotation are responsible for the distortion of oxygen octahedrons and off-center shift of Bi atoms, both of which result in the local structural distortion. Based on high-pressure transport property measurements, the local electron habitation and interaction are tightly correlated to local structure distortion.

## Methods

The synthesis detail of polycrystalline Bi(Ni_1/2_Ti_1/2_)O_3_ can be found in our previous work^11^. Briefly, raw materials of Bi_2_O_3_, NiO and TiO_2_ were mixed in a 1:1:1 molar ratio and calcinated at 5 GPa and 1273 K for ~30 min in a cubic anvil-type pressure apparatus. The total X-ray scattering under high pressure (up to ~30 GPa) was conducted at X17-B3 beamline of National Synchrotron Light Source (NSLS), Brookhaven National Laboratory (BNL). The X-ray energy was tuned to 81.087 keV, which corresponds to the wavelength of 0.15290 Å. A short symmetric diamond anvil cell (DAC) with diamonds of 500 μm culet size was used in the scattering experiment with a downstream opening angle of ~60°. A tungsten gasket was pre-indented to ~50 μm, and a hole of 250 μm diameter was drilled to serve as sample chamber. A background scattering was collected on DAC plus empty gasket at ambient pressure, and was applied as a background correction for measurements performed under high pressure. The Bi(Ni_1/2_Ti_1/2_)O_3_ powders were then loaded into the sample chamber, along with a piece of ruby pressure marker. The program Fit2D^18^ was used to integrate the raw intensity images, and the program PDFgetX2^19^ was used for data treatment. The integrated scattered intensity was corrected for background, Compton scattering, and absorption, and was then converted to total scattering structure function. The program PDFgui^20^ was used for PDF modeling and structural refinement using the X-ray PDF data range from 1–80 Å. The experimental PDFs were obtained by a Fourier transform of the total scattering structure function up to Q_max_ of 20 Å^−1^, and resolution of the instrument, including Q_damp_ = 0.031729 and Q_broad_ = 0.020608, was determined with cerium oxide powder (CeO_2_) measured under the same conditions. Structural parameters for Bi(Ni_1/2_Ti_1/2_)O_3_ were obtained from Ref. 11 as starting point in PDF refinements. During the fit we varied the scale factor, the unit-cell, the x- (y-, and z-) coordinate of Bi (Ni and Ti) , and the isotropic thermal displacements (U_ij, ij = 11,22,33_) for all atoms in Bi(Ni_1/2_Ti_1/2_)O_3_ phase (all constrained by the *Pn*2_1_*a* space group). The r-dependence of the Bi(Ni_1/2_Ti_1/2_)O_3_ PDF peak width due to correlated thermal motions was refined using the variable delta1 (δ1). Illustration of the fits for a selected *r* range is shown in Fig. 1.

High-pressure resistivity measurement up to ~30 GPa was conducted using the standard four-probe method in a symmetric DAC. A thin layer of hexagonal boron nitride was employed as insulation between the T301 stainless steel gasket and the four platinum leads. A Keithley 6221 current source, 2182A nanovoltmeter and 7001 switch system were used for the resistance measurement. A Raman spectrometer equipped with a 532 nm laser and 1200-grinding at high pressure synergetic consortium (HPSynC), Argonne National Laboratory, was used to study vibrational properties in a symmetric DAC. The sample images under different pressures were recorded by a Leica microscope with the same luminance light.

## Additional Information

**How to cite this article**: Zhu, J. *et al.* Local structural distortion and electrical transport properties of Bi(Ni_1/2_Ti_1/2_)O_3_ perovskite under high pressure. *Sci. Rep.* **5**, 18229; doi: 10.1038/srep18229 (2015).
